# Survival from kidney cancer in England and Wales up to 2001

**DOI:** 10.1038/sj.bjc.6604602

**Published:** 2008-09-23

**Authors:** B W Hancock

**Affiliations:** 1 Cancer Research Centre, Weston Park Hospital, Whitham Road, Sheffield S10 2SJ, UK

## Clinical presentation and diagnosis

Renal cell carcinoma is by far the commonest cancer in this group. Presentation may be with local symptoms. Thus haematuria, usually painless though sometimes with colicky pain secondary to blood clots, is common. Less often, a swelling or aching in the loin may be noticed by the patient. Often and increasingly the tumour is picked up coincidentally during an imaging investigation (e.g., ultrasound scan of the abdomen). Metastatic disease may be evident, in up to a third of patients, as bone, lung or brain secondaries or by systemic features such as weight loss, fever, malaise and anaemia. Such features may also be present with organ-confined disease.

## Treatment

Surgery is the cornerstone of treatment. Nephrectomy is potentially curative for tumours confined to the kidney and/or regional nodes and is of value in reducing tumour bulk in palliating advanced disease. With metatastic disease radiotherapy may help in palliating local symptoms. Conventional chemotherapy has little value and although immunotherapy with interferon-*α* or interleukin-2 does marginally improve progression-free and overall survival, it is at the cost of marked toxicity. However international efforts continue to develop ways of identifying the small minority of patients who will benefit greatly from immunotherapy. More recently knowledge of molecular pathways involved, particularly in clear cell renal carcinoma, has provided a rationale for targeted treatment. Agents have been identified, which target gene products, such as vascular endothelial growth factor (VEGF) and platelet derived growth factor (PDGF), implicated in tumourigenesis. Such agents have demonstrated significant activity in clinical trials and represent a major advance in treatment (Brugarolas, 2007).

## Incidence and survival patterns

Only a small number of kidney cancers have an inherited basis, as for example von Hippel–Lindau (VHL) disease where the cancer risk for a variety of tumours including renal carcinoma is transmitted in an autosomal dominant manner. It is now known that the VHL suppressor gene is mutated or silenced in most clear cell renal carcinomas though the initiator(s) of this event are still unknown. Information on risk factors for renal cell carcinoma has come from a large number of international case control and cohort studies (McLaughlin *et al*, 2006). Cigarette smoking and obesity are the most consistently established causal factors, smoking having a greater effect in men, obesity possibly in women. It is important to take these factors into account when interpreting incidence trends. Across the community obesity is increasing and smoking decreasing. From the data presented by Westlake *et al* (2008), there has been an improvement in relative survival over the period 1986–1999. Most likely this reflects general improvements in health care, and possibly earlier diagnosis, rather than in specific therapies – the 1980s and 1990s were the decades of biological therapy where early promise of improvements in survival never came to significant fruition. Deprivation still seems to be an important factor in prognosis in men, but less so in women; the reasons for this are unclear. Certainly these trends cannot be related to known changes in smoking and obesity but could be linked to changes over time in health care and earlier diagnosis across the socioeconomic spectrum.

## Future clinical implications

It is disappointing that survival figures for English patients (up to mid 1990s), were among the lowest in Europe but reassuring that improvements have particularly been seen during the late 1990s. All patients now have the benefit of having their case discussed by a multidisciplinary team including specialist urology and oncology clinicians, with access to optimal treatments and also information on clinical trials. For example, a new randomised study (SORCE) assessing the function of adjuvant sorafenib (one of the new generation of VEGF inhibitors) in high-risk resected renal cancer has recently been launched. For metastatic disease sunitinib and sorafenib (VEGF inhibitors) have now been licensed in the United Kingdom on the basis of evidence from large randomised controlled clinical trials (Motzer *et al*, 2007; Escudier *et al*, 2007). These agents and others are not yet generally available in the National Health Service and are currently being appraised by the National Institute for Clinical Excellence. There is every hope, therefore, that the improvements in survival reported in the accompanying article will continue into and beyond the first decade of the 21st century. In addition there is considerable interest in prognostic models (based on clinical and laboratory data) that have been constructed to provide meaningful risk stratification for clinical trials (Shuch *et al*, 2006), the results of which can then be extrapolated to tumour registry survival data and help in our understanding of the factors that influence trends and socioeconomic inequalities in survival.
